# Ultrasonography-Guided Perineural Injection of the *Ramus ventralis* of the 7 and 8th Cervical Nerves in Horses: A Cadaveric Descriptive Pilot Study

**DOI:** 10.3389/fvets.2020.00102

**Published:** 2020-02-25

**Authors:** Gwenola Touzot-Jourde, Olivier Geffroy, Amélie Tallaj, Olivier Gauthier, Jean-Marie Denoix

**Affiliations:** ^1^INSERM, UMR 1229, Regenerative Medecine and Skeleton, University of Nantes, ONIRIS, Department of Veterinary Clinical Sciences, Nantes, France; ^2^INRA/ENVA, UMR 957, Biomechanics and Equine Locomotor Pathology Research Unit, Centre for Imaging and Research in Equine Locomotor Disorders (CIRALE), National Veterinary School of Alfort, Maisons-Alfort, France

**Keywords:** horse, ultrasonography, cervical, nerve roots, anesthesia, analgesia

## Abstract

**Objective:** To describe the feasibility and dye diffusion of selective perineural injection of the 7 and 8th cervical nerve (C7 and C8) *ramus ventralis* under ultrasonographic guidance in horses.

**Study design:** Prospective experimental pilot cadaver study.

**Animals:** Four equine cadavers of similar body weight (420–480 kg) and neck conformation.

**Methods:** Five C7 and five C8 *rami* were perineurally injected with a dye solution. Anatomic dissections including vertebral canal opening were conducted to confirm nerve dye staining and describe the extent of color diffusion.

**Results:** The *ramus ventralis* of the spinal cervical nerves was visualized in all cadavers. All the injections were successful in staining a portion of the nerve trunk. Eight *rami* had a uniform transversal staining of the nerve trunk that longitudinally covered a distance >2 cm. One C7 and one C8 nerve trunk showed incomplete transversal staining with a more concentrated color on its half cranial aspect and a longitudinal coverage of <2 cm. Five injections resulted in dye extending proximally and medially into the epidural space. Volume had no appreciable effect on the extent of nerve staining. A greater proportion of epidural diffusion was found with injections done within less than one cm distally to the articular processes. All injections were considered to be selective for the targeted nerve.

**Conclusion and clinical relevance:** Ultrasonography-guided perineural injection of C7 and C8 *ramus ventralis* is a feasible technique that may have multiple applications in multimodal analgesia in horses. Further clinical study will be necessary to determine the appropriate drug, dosage, and volume to inject and to confirm its usefulness.

## Introduction

Arthropathy of the articular process joints of the caudal cervical spine has been associated with neck pain and stiffness as well as forelimb lameness in horses (1–3). Enlarged articular process joints with periarticular bony proliferation and associated capsule effusion have been documented to reduce intraforaminal space resulting in possible nerve root impingement (1, 4–6). Compressive neuropathy of the 7th cervical nerve root has been evidenced histologically on post-mortem specimens in horses (1, 2).

Cervical radiculopathy is considered to be a debilitating disease in humans with variable clinical signs that range from pain, numbness, and/or tingling in the upper extremity to electric shock type pain and muscle weakness (7). In dogs, the pathognomonic clinical presentation is the nerve root-signature sign, which is similar to the signs seen in horses. Dogs adopt a non-weight bearing posture on the affected side, holding their forelimb in a semi-flexed position. They may also show some signs of neck pain (8).

Magnetic resonance imaging (MRI) and contrast-enhanced computed tomography (CT) are the imaging techniques of choice to identify articular process enlargement, intervertebral foramen size reduction, and nerve root impingement (6, 7, 9, 10). In horses, despite recently improving technology, these imaging techniques are still of limited clinical applicability to the equine caudal cervical region due to technical constraint imposed by the relative size of the equine neck/chest/shoulders compared to the instrument bore.

Selective cervical nerve root injections have been used in humans and dogs mainly for therapeutic purposes and, less commonly, to confirm the site of pain (8, 11–16). Periradicular injection techniques have been developed under fluoroscopic guidance but more recently ultrasonography-guided injections have been shown to be as effective and possibly safer in humans (11, 14, 16, 17). More precisely, transforaminal injections around the cervical nerve root are classically performed using the fluoroscopic techniques while ultrasonographic guidance has allowed for more distal injections around the *ramus ventralis* of the spinal nerve (16). Scientific reports on both techniques typically use the term “nerve root” for both parts of the spinal nerve. According to the *Nomina Anatomica Veterinaria* (2012), the nerve segment considered in this paper is the *ramus ventralis*.

Ultrasonography (US) is commonly used in horses for perineural/periarticular/intraarticular blocks and therapeutic infiltrations (18–21). Periarticular and intraarticular corticosteroid injections of the articular process joints have been reported to possibly improve clinical signs related to articular process arthropathy (19). Due to its clinical availability, applicability, and safety while also taking into consideration imaging techniques used in humans and their possible applicability in horses, US should constitute the guidance of choice to perform a selective caudal cervical nerve root injection in horses (16, 19, 22).

The *ramus ventralis* of the 7 and 8th cervical spinal nerve (C7, C8) with the 1st thoracic (T1) provides the largest roots to the equine brachial plexus with a minor contribution of the C6 and T2 nerves (23). The aim of this pilot study on cadavers was to describe the feasibility and the dye diffusion of selective perineural injection of C7 and C8 *ramus ventralis* under ultrasonographic guidance in horses.

## Materials and Methods

The experimental protocol was reviewed and approved by the Ethical Committee for Animal Research of the ANSES/ENVA/Paris-Est Créteil University (authorization number COMETH 16-025). Four cadavers of recently euthanized horses for reasons unrelated to the study were used (storage <36 h at 4°C). They were 3 to 5 year old, 420 to 480 kg French Trotters with a similar lean neck conformation. A radiographic screening including the cervical region was available in each individual medical file. No abnormalities were found on the lateral radiographic view of their caudal cervical region (5th cervical to 1st thoracic vertebrae, C5-T1).

### Ultrasonographic Technique and Landmarks

**Figure 1:**
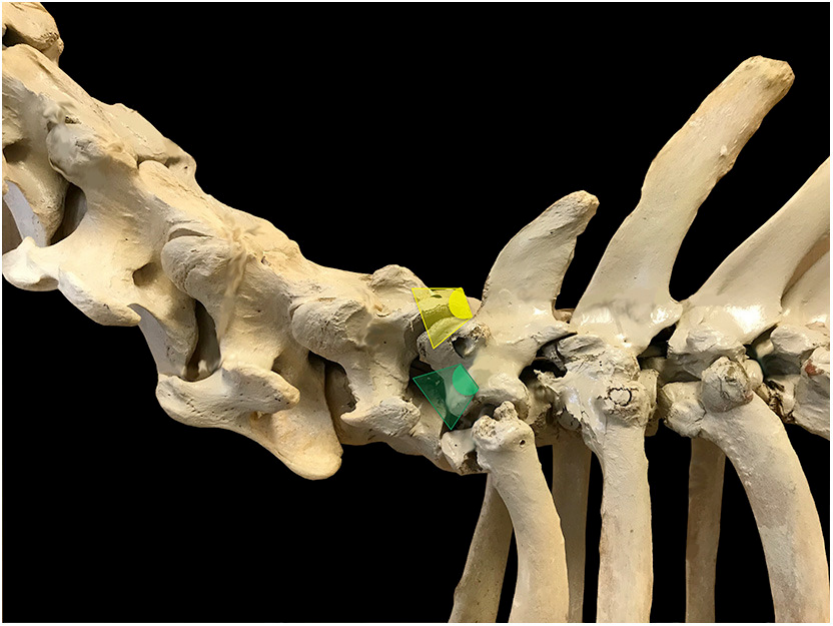
Ultrasound probe positioning and orientation to visualize C8 *ramus ventralis* in a longitudinal section in horses. The probe is initially oriented perpendicular to the vertebral axis to obtain a reference image of the 2 articular processes and the joint space (yellow scan field) then glided ventro-caudally with an approximate 20° angle from its initial position (counter-clockwise rotation on the left side of the neck, green scan field).

**Figure 2:**
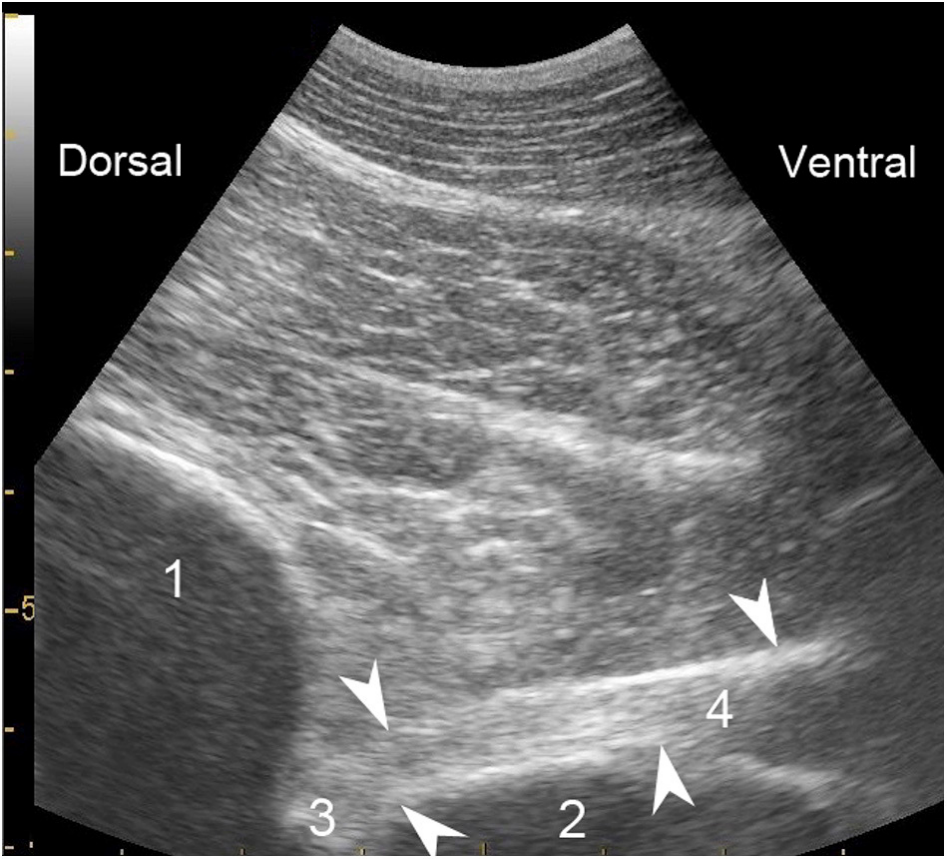
Representative longitudinal ultrasonographic section of C8 *ramus ventralis* in an equine cadaver. Ventrally to T1 cranial articular process, the nerve trunk (arrows) shows echogenic longitudinal fasciculi bordered by a more echogenic sheath. 1- Cranial articular process of T1; 2- Vertebral body (margin of the fossa) of C7; 3- Intervertebral foramen; 4- *Ramus ventralis* of C8.

The cadavers were positioned in lateral recumbency with the side to be injected up and the uppermost forelimb retracted caudally. The cervical vertebrae C6 and C7 were located using manual palpation of the transverse process of C1 and counting segments of the width of the hand in a caudal direction as described by Mattoon et al. (18). After adequate skin preparation (hair clipping and surgical skin disinfection), a micro-convex 5–8 MHz ultrasound probe (C11x, SonoSite Edge portable ultrasound machine, FUJIFILM SonoSite France SARL) was applied to the most caudal segment to identify the C7-T1 articular process joint. The probe was oriented perpendicularly to the vertebral axis to obtain a reference image of the 2 articular processes and the joint space (22). Then to visualize a longitudinal section of C8 *ramus ventralis*, the probe was glided ventro-caudally with an approximate 20° angle from its initial position by orienting the uppermost edge of the probe cranially and ventrally (counter-clockwise rotation on the left side of the neck) (Figure 1). The probe was then glided along the nerve course over the vertebral body between the articular process and the transverse process to identify the best *ramus ventralis* longitudinal section image possible that would allow for easy needle placement and product injection monitoring on the US screen (Figure 2). A 88 mm 20G spinal needle (Spinocan, BBraun France SA) was inserted ~2 cm caudo-ventrally to the transducer and advanced “in plane” until the needle tip was <5 mm adjacent to the nerve surface. The injection of the syringe content was monitored on the US screen. The same technique was repeated one segment cranially on the C7 *ramus ventralis*. Localizing the best injection window by US was conditional on individual horse neck conformation and ability to optimize the cadaver neck to forelimb and shoulder position. As a result the point of injection along the *ramus ventralis* course was not standardized as a set distance from the articular processes. Therefore, the distance between the articular process ventral border and the needle tip was recorded. The injection was qualified as proximal if the distance was <1 cm or distal if it was within 1 to 2 cm.

### Dyes and Dye Solution

Methylene blue powder (Bleu de méthylène, RAL Diagnostics, France) was used in the first three horses and reconstituted as a 0.12% solution. Following the analysis of the first results with methylene blue staining and recent scientific information on dye nerve staining (24, 25), an additional cadaver was injected with multiple color permanent marking tissue dyes (Davison Marking System; Bradley Products, Inc., MN, USA) at a 1:50 dilution (1 ml of dye in 50 ml of carrier solution).

For both dyes, the solution carriers were chosen to reproduce as much as possible an *in vivo* situation and a foreseen clinical study (25, 26). The solution consisted of one part iohexol (Omnipaque 300 mg/ml, GE Healthcare SAS, France) for 2.5 parts lidocaine 2% (Lidocaïne sans conservateur 20 mg/ml, Laboratoire Aguettant, France). Carrier agent respective proportions were extrapolated from similar studies (16, 26).

### Dye Injection Volume and Allocation

The initial objectives of perineural injections with methylene blue solution were to observe tissue dye staining and test the injection selectivity for the *ramus ventralis* considering possible diffusion to the adjacent cranial and/or caudal ispilateral *ramus* in addition to diffusion to the epidural space across midline. Three equine cadavers stored for <36 h at 4°C were used after being at room temperature for 3 h. They received unilateral perineural *ramus ventralis* injections of C7 and C8 with the methylene blue solution (6 injections *in toto*). The use of a single dye color limited the number of injection possibilities and testing conditions. As a result and to facilitate cadaver handling and dissection, it was decided to test 2 volume conditions on the same side of the neck. While considering the *ramus ventralis* size difference (width of C8 1.5 to 2 times larger than C7), the two conditions tested were C7–7 ml/C8–14 ml on two horses (horse 1 and 3) and C7–7 ml/C8–7 ml on horse 2. Perineural injection volumes selected (7 and 14 ml) were extrapolated from similar studies using an allometric scale (12, 16, 26). Left and right neck injections and condition assignment for the 3 cadavers were randomly selected using a closed envelope technique.

Following the results on the 3 methylene blue cadavers and to confirm the findings, an additional cadaver received, four injections with a solution of permanent marking tissue dye within 30 min from euthanasia. The use of 4 different colors allowed to perform bilateral injections without compromising the evaluation of individual injection selectivity for each *ramus ventralis*. Opposite randomized conditions were tested: C7–7 ml/C8–14 ml on the left and C7–14 ml/C8–7 ml on the right.

### Dissection and Nerve Staining Evaluation

The injections in the 3 methylene blue cadavers were performed one cadaver after the other on the same morning and followed by the dissection respecting the cadaver injection sequence within 1 to 3 h after the injections. Horse 4 was dissected within the same time frame, respecting the injection side order (right then left). After removing the ipsilateral forelimb and the cervical muscles attached to it, the C7 and C8 *ramus ventralis* were isolated from the surrounding muscles along their course from the articular processes to the brachial plexus. The vertebral canal was opened *in situ* on horse 1, 2, and 4. On horse 3 (C7–7 ml/C8–14 ml) in an attempt to better visualize possible dye spread to the vertebral canal, the C5 to T2 vertebral segment was isolated as one block for frozen transverse sections.

The distribution and extent of nerve staining were evaluated cranio-caudally for partial or complete transversal coloration across the nerve trunk and longitudinally, considering a staining length greater than 2 cm to be indicative of what would be a clinically effective nerve blockade (27). For horse 1, 2, and 4, epidural/subarachnoid diffusion was assessed upon opening the vertebral canal looking at the ventral and dorsal spinal nerve roots as well as the meninges around the spinal cord. The meninges were then transected longitudinally on their dorsal sagittal aspect and reclined to inspect for presence or absence of dye on the spinal cord, the spinal nerve rootlets and the arachnoid membrane. The assessment on horse 3 was done through observation of 1 cm thick transverse sections of the vertebras from C5 to T2.

## Results

**Figure 3:**
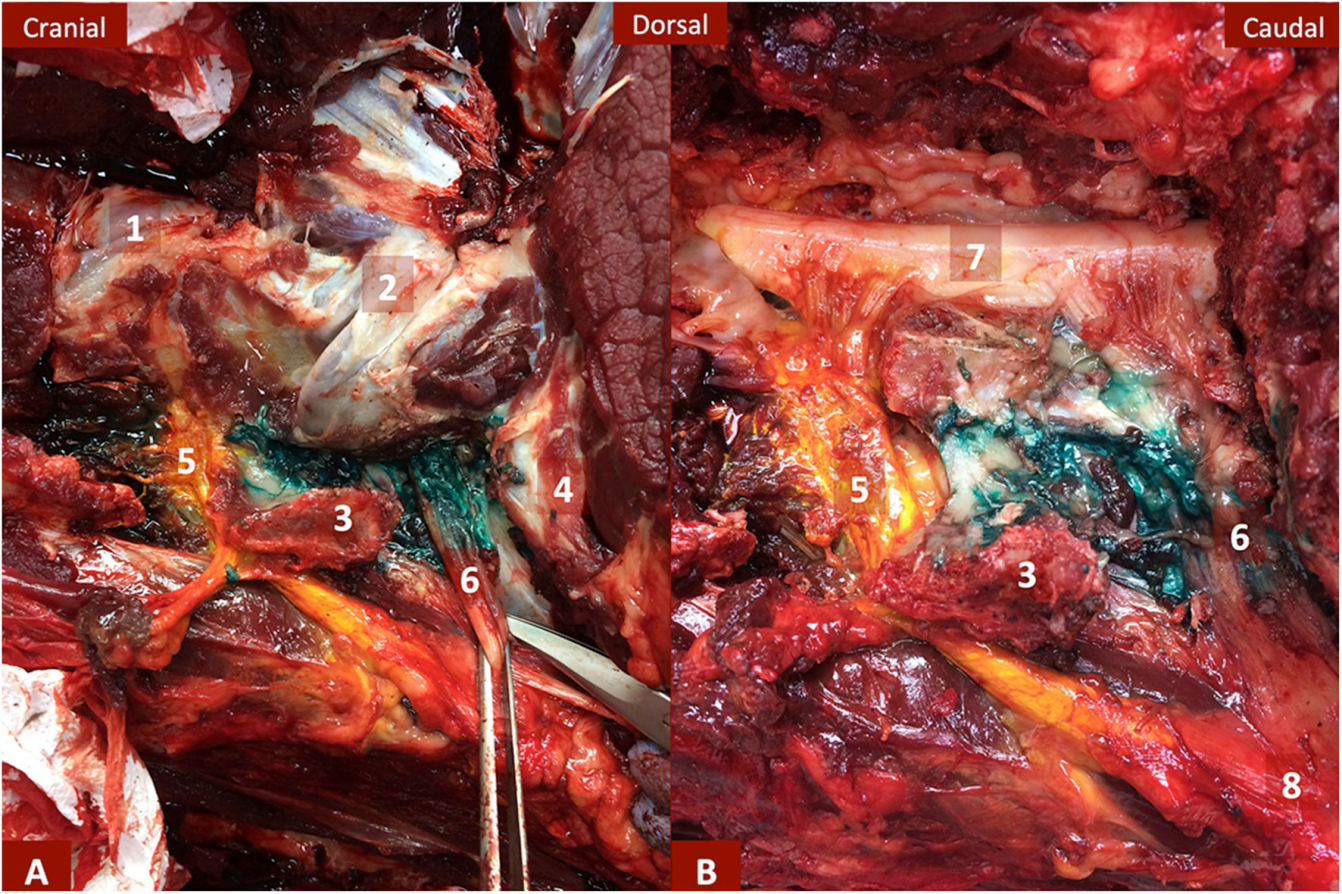
**(A,B)** Dissection of the left C7 and C8 *ramus ventralis* of horse 4 after removal of the neck muscles **(A)** and after opening of the vertebral canal **(B)**. C7 has an extensive yellow staining reaching the epidural space and the meninges and spreading to the limit of the brachial plexus. C8 green dye spread is delimited to the ventral aspect of the articular processes with dye diffusing dorsally to C7 transverse process. 1- Caudal articular process of C6 and cranial articular process of C7; 2- Caudal articular process of C7 and cranial articular process of T1; 3- Transverse process of C7; 4- First rib; 5- *Ramus ventralis* of C7; 6- *Ramus ventralis* of C8; 7- Spinal cord covered by the meninges; 8- Brachial plexus.

The *ramus ventralis* could be identified by US in all cadavers. Cervical segment identification was erroneous on one methylene blue cadaver (horse 1), leading to C6 and C7 *ramus ventralis* injections instead of C7 and C8. The 10 performed injections corresponded to 1 C6–7 ml, 3 C7–7 ml, 2 C7–14 ml, 2 C8–7 ml, and 2 C8–14 ml. Results of individual cadaver perineural *ramus ventralis* injections are presented in Table 1. The probe positioning adjustment to obtain an optimized *ramus ventralis* visualization for the injection resulted in 5 injections that were qualified as proximal and 5 as distal. All the injections were successful in staining a portion of the nerve trunk and were identified as selective for the *ramus* concerning their distal diffusion toward the brachial plexus. Eight *ramus ventralis* had a uniform complete transversal staining of the nerve trunk that longitudinally covered a distance >2 cm, of which three *ramus* (2 C7–7 ml, horse 2 and 4; 1 C8–14 ml, horse 3) were colored from the articular processes to the limit of the brachial plexus (Figure 3A). One C7–14 ml and one C8–7 ml *ramus ventralis* (horse 4 and 2 respectively) showed incomplete transverse staining with a more concentrated color on their cranial half and a longitudinal coverage of <2 cm.

**Table 1:** Caudal cervical *ramus ventralis* perineural injections on 4 equine cadavers: injection condition allocation (neck side and injection volume), injection site along the nerve course, dye choice, and extent of diffusion.

**Cadavers**	***Ramus Ventralis*** **Left (L) or right (R) side** **Volume injected**	**Injection site**	**Length of nerve staining**	**Epidural diffusion**
**Methylene blue dye**				
Horse 1	C6 L 7 ml	Distal	>2 cm^*^	No
Horse 1	C7 L 14 ml	Proximal	>2 cm	Yes(+ sub-arachnoid)
Horse 2	C7 L 7 ml	Distal	>2 cm^*^	No
Horse 2	C8 L 7 ml	Proximal	<2 cm^**^	Yes
Horse 3	C7 R 7 ml	Proximal	>2 cm	Yes (frozen sections)
Horse 3	C8 R 14 ml	Distal	>2 cm^*^	Yes (frozen sections)
**Permament marking tissue dye**				
Horse 4	C7 R 14 ml	Proximal	<2 cm^**^	No
Horse 4	C8 R 7 ml	Distal	>2 cm	No
Horse 4	C7 L 7 ml	Proximal	>2 cm^*^	Yes
Horse 4	C8 L 14 ml	Distal	>2 cm	No

* *Longitudinal staining of the ramus ventralis from the ventral border of the articular processes to the limit of the brachial plexus*.

** *Incomplete transversal staining with more concentrated color on cranial half of the nerve trunk*.

On horse 3, the *ramus ventralis* staining from the articular processes to the brachial plexus was evaluated *in situ* as with on the other cadavers but the potential epidural spread was investigated by transverse sections. Five days of freezing were required to obtain a completely frozen vertebral segment. Indeed, a first attempt at slicing the cranial edge of the isolated vertebral segment (C6 vertebra) on day three showed an incompletely frozen segment core preventing completion of the trans-section. The transverse sections presented an extensive methylene blue diffusion into the vertebral canal reaching proximal and distal vertebral segments (C6-T1) and crossing midline. Meninges, venous sinuses and surrounding vertebral bone were deeply colored. The extensive diffusion was attributed to the methylene blue propensity to diffuse in tissue over time associated with the slow freezing process (24). The evaluation technique was therefore not pursued but the data was included in the epidural spread evaluation as time would be clinically relevant.

Overall, the effect of volume was not clearly recognizable on nerve length staining nor on epidural spread likelihood. Four out of five proximal injections resulted in an epidural spread (Figure 3B), of which one showed a mixed epidural/subarachnoid diffusion, compared to only one out of five distal injections that happened to be evaluated by the frozen section. Apart from an epidural spread in the vertebral canal across midline and to the neighboring segments on the frozen sections, no dye stain was evidenced neither on the opposite side nerve nor on the proximal or distal nerve. Therefore, all injections were considered to be nerve selective regarding their proximal diffusion in the vertebral canal.

## Discussion

This study evaluated the nerve coloration extent of ultrasonographically guided C7 and C8 *ramus ventralis* perineural injections in horses. The technique was effective in staining the entire width of the nerve trunk on a length >2 cm in eight out of ten injections.

Ultrasonographic visualization of the C7 and C8 *ramus ventralis* was easily achieved with a micro-convex 5–8 Mz US probe and allowed for adequate needle positioning. Some degree of *rigor mortis* may have precluded from ideal neck/foreleg positioning on horse 1 and, associated with the cervical segment identification method, resulted in injections of C6-C7 *rami* instead of C7-C8 *rami*. More precise methods have been described such as the use of external radio-opaque markers for cervical radiographs before US or US identification of the deep cervical artery passing caudo-dorsally to C7-T1 articular process joints (3). These methods were not applicable to the experiment settings but would be easily undertaken on standing horses in a clinical setting.

The injection site was selected individually for each nerve upon obtaining the best US visualization of the nerve trunk. As a result, needle tip positioning over the nerve trunk was close to the articular processes in five of the total ten injections, of which four out of five were C7 injections. The anatomic conformation of the C6 vertebral transverse process with a marked dorsal tubercle prevented C7 *ramus ventralis* distal portion visualization and may have contributed to the proximal injections. Due to its large size and width, C8 *ramus ventralis* was the easiest to identify in longitudinal section for an in plane US-guided injection (23). It resulted predominantly in distal injections.

The length of nerve staining >2 cm was compatible with a clinically effective blockade of nerve conduction for eight injections, of which 50% resulted in staining of the complete nerve trunk length from the articular processes to the brachial plexus (27). The absence of an identifiable volume effect has to be linked to the small sample size and the study design, main limitations of this pilot study. Interestingly, Anderberg et al. (26), while testing 3 different volumes of injection in human cadavers, found that the length of nerve staining was not correlated to the volume.

The change in epidural spreading assessment from *in situ* dissection to evaluation on frozen transverse sections may appear as a lack of protocol standardization. It was initially thought to be more adapted to visualize the entire vertebral canal and spinal cord compared to an *in situ* canal opening by a lateral window. Unexpectedly, the intense vertebral canal tissue coloration up to cortical bone, its extent across midline and to the cranial and caudal vertebrae rendered necessary to evaluate the choice of methylene blue as a dye and the effect of time on its diffusion ability in tissue.

Methylene blue has been used for nerve staining to identify nerve structures during surgery or for neurohistology as well as to document perineural injection techniques in animal cadaveric studies (16, 23, 27). Even though it is rarely mentioned in cadaveric studies, time has an effect on its extent of diffusion; this was evidenced in organ pharmacokinetics studies in rodents (24). The compound is a water-soluble cation but, at physiologic pH, it is present in tissue mostly in its reduced non-ionized lipid-soluble colorless form that has all the physical properties for extensive diffusion across tissue and cellular membrane. Moreover, exposure of dye impregnated tissue sections to air results in appearance of the blue color by oxidation. These facts may bring some explanation to the present study results, especially to the extended diffusion into the vertebral canal identified on the delayed frozen sections. The anatomic piece took more than 3 days to freeze at −20°C. It is possible to postulate that a faster freezing process may have limited this effect. It was also witnessed during the immediate cadaver dissection that the blue color spreading had a tendency to increase over time, was easily transferable to gloves and surrounding tissues if careful tissue manipulation was not applied. Similarly, the assessment of the saw sliced frozen vertebral segments allowed seeing very minimal methylene blue “smudging” but manipulation of the sections over time showed dye spreading to surrounding tissues.

The impact of dye diffusion properties and dissection approach on the assessment of an injection technique has been discussed by Portela et al. (25). Taking into account their findings and the present study results with methylene blue, it was decided to perform four more injections with the dye used by the authors, a permanent marking tissue dye designed to permanently mark tissue in less than 5 min and orient tissue specimen for surgery or histology. Different colors are available and allowed testing for injection selectivity using a single cadaver that therefore received 4 *ramus ventralis* perineural injections (2 C7 and 2 C8). No further expansion of the dyes was appreciable during the dissection. Used in the present study setting, it is hypothesized that the permanent marking tissue dye more accurately tested for adequate needle placement and volume diffusion compared to methylene blue. Comparison of the dissection process and dye behaviors over time during tissue manipulation lead to the thought that, with the permanent marking tissue dye, the stained area was mainly the result of the needle placement, the volume/pressure of the injection and the tissue reaction. The intrinsic diffusing property of the dye was only linked to the dilution solution and not to the dye *per se*. It may however represent an underestimation of drug diffusion. Nonetheless, the results on the last cadaver confirmed methylene blue findings concerning staining extent of the nerve. The injection of 7 and 14 mL with both dye solutions resulted mostly in a greater than 2 cm length staining. To confirm the suitability of the permanent marking tissue dye, further dissections reproducing the study setting including frozen section assessment are needed as well as evalutating the impact of the freezing process duration.

Apart from the delayed epidural spread on the frozen section, all the injections were considered nerve selective but may not exactly reflect drug diffusion over time in living horses. On four injections, the dye reached the limit of the brachial plexus or diffused cranio-caudally over the transverse process nearly reaching the adjacent *ramus ventralis* (2 dyed with methylene blue and 2 with permanent marking dye). It can be hypothesized that, with more time between the injection and the dissection, nerve selectivity would have been lost. Even though the used dye carrier solution aimed at reproducing a clinical setting, it cannot be certain that drugs would not reach the contiguous *ramus ventralis* or would not spread as much due to systemic resorption by the lymphatic and venous system.

Of the 10 perineural injections performed all cervical nerves considered, five injections resulted in a transforaminal diffusion into the epidural space. Four of those 5 were C7 injections and were qualified as proximal. One reached the subarachnoid space and happened to be the injection done the closest to the articular process (less than 5 mm from the articular process ventral border). Similar findings have been reported by Yamauchi et al. (16) that advocate the advantage of ultrasonographic guidance to perform an extraforaminal perineural injection without epidural spreading. The safety of transforaminal injections in humans has been questioned following recognition of major complications after accidental intradural or intra-arterial injections (17, 26, 28, 29). The study was undertaken on equine cadavers so the risk of intravascular injection and arterial wall damage could not be fully tested. Recommendations to limit the risk of complications include injecting along the nerve root distally to the articular process with a dorsal approach to the nerve, the use of US to allow for the identification of vulnerable vessels, and the use of non-particulate steroids; the absence of epidural spread in US-guided perineural corticosteroid injections have not been linked to a decreased efficacy compared to transforaminal injections in human radicular pain (16, 17).

In conclusion, the perineural injection of C7 and C8 *ramus ventralis* using ultrasonographic guidance may represent a feasible technique for local perineural drug delivery in horses. The results of this study suggest that a proximal injection along the nerve may favor a transforaminal diffusion reaching the epidural space while a more distal injection may target the *ramus ventralis* on its course over the vertebral body. The volumes (7–14 ml) appear adequate but need to be confirmed in living horses. Clinical applicability and efficacy still have to be investigated.

## Data Availability Statement

All datasets generated for this study are included in the article/supplementary material.

## Ethics Statement

The study was reviewed and approved by the Ethical Committee for Animal Research of the ANSES/ENVA/Paris-Est Créteil University (authorization number COMETH 16-025).

## Author Contributions

GT-J and J-MD conceived the study design. GT-J, J-MD, and AT participated in data collection and analysis. GT-J, J-MD, OGe and OGa contributed to the writing of the manuscript.

### Conflict of Interest

The authors declare that the research was conducted in the absence of any commercial or financial relationships that could be construed as a potential conflict of interest.
